# A Queer Incident

**Published:** 1888-03

**Authors:** Amarala Martin

**Affiliations:** Cairo, Ill.


### A QUEER INCIDENT.

*To the Editor of* HALL'S JOURNAL OF HEALTH:

SIR—I am possessed of a wonderful healing power which those who witness may call magnetism, hypnotism, psychic force, or anything else most agreeable to themselves. The fact of the power remains the same call it what they may.

One day, about three years ago, a strange lady (whom I will call Mrs. B.) sent for me to come to her immediately as she was very ill. I was not a professional, and was, therefore, rather surprised at the message, but hastened to the place mentioned by the child who came for me.

It seemed that Mrs. B. had been suffering greatly for a few days with inflammatory rheumatism and fever, and to this was suddenly added a stroke of partial paralysis. I found her attendants trying to bathe the affected parts with liniment, but their every touch was followed by a cry of agony. As I approached Mrs. B. the expression of her face was one of surprise and something akin to fear, but it soon changed into one of earnest appeal. I took her hands in mine, speaking some words of encouragement, and under my touch she was entirely free from pain in less than an hour.

On the following day I called and found her able to walk about the house and yard and feeling quite comfortable. She then told me why she sent for me and why she felt surprised when I entered the room. "Yesterday, when I was suffering the most excruciating pain," said she, "I saw a lady,—or vision, as I now believe—come to my bedside, and she told me to send for you and you would cure me. When you came I saw that you were the exact counterpart of the image, vision, spirit or whatever it was, that had just visited me. Now, I do not believe in any nonsense or new-fangled ideas about spirits, for I am an out and out Christian, and belong to the Baptist church, but I hope to die this minute if I didn't see you—or *it*—just as I tell you."

At the hour Mrs. B. claimed that she saw me I was at home, and neither asleep, unconscious or aware of any unusual sensation. And have no power of which I am aware, of sending my visible spirit on errands of mercy. How was it, then ?

Cairo, Ill. AMARALA MARTIN.

